# Fstl1 Antagonizes BMP Signaling and Regulates Ureter Development

**DOI:** 10.1371/journal.pone.0032554

**Published:** 2012-04-02

**Authors:** Jingyue Xu, Xin Qi, Jianfeng Gong, Mingyan Yu, Fangxiong Zhang, Haibo Sha, Xiang Gao

**Affiliations:** 1 MOE Key Laboratory of Model Animal for Disease Study, Model Animal Research Center, Nanjing University,Nanjing, China; 2 Zhejiang Provincial Key Lab for Technology and Application of Model Organisms, School of Life Sciences, Wenzhou Medical College, Wenzhou, China; 3 Institute of Neuroscience, Shanghai Institute of Life Sciences, Chinese Academy of Sciences, Shanghai, China; National Cancer Center, Japan

## Abstract

Bone morphogenetic protein (BMP) signaling pathway plays important roles in urinary tract development although the detailed regulation of its activity in this process remains unclear. Here we report that *follistatin-like 1* (*Fstl1*), encoding a secreted extracellular glycoprotein, is expressed in developing ureter and antagonizes BMP signaling activity. Mouse embryos carrying disrupted *Fstl1* gene displayed prominent hydroureter arising from proximal segment and ureterovesical junction defects. These defects were associated with significant reduction in ureteric epithelial cell proliferation at E15.5 and E16.5 as well as absence of subepithelial ureteral mesenchymal cells in the urinary tract at E16.5 and E18.5. At the molecular level, increased BMP signaling was found in *Fstl1* deficient ureters, indicated by elevated pSmad1/5/8 activity. *In vitro* study also indicated that Fstl1 can directly bind to ALK6 which is specifically expressed in ureteric epithelial cells in developing ureter. Furthermore, Sonic hedgehog (SHH) signaling, which is crucial for differentiation of ureteral subepithelial cell proliferation, was also impaired in *Fstl1^-/-^* ureter. Altogether, our data suggest that Fstl1 is essential in maintaining normal ureter development by antagonizing BMP signaling.

## Introduction

Congenital malformations of the kidney and urinary tract are the primary causes of renal failure in children and young adults [1] and frequently affect human infants. Many of these hereditary diseases display hydroureter and/or hydronephrosis with dilatation of the ureter and/or the renal pelvis, caused by failure to conduct urine from the renal pelvis to the bladder [2], [3]. The underlying causes of these congenital malformations are still largely unknown.

Murine urinary tract development is a model that is broadly used to understand the underlying mechanism of human urinary tract malformations. On gestational day 10.5 (E10.5), ureteric bud, an epithelial outgrowth from Wolffian duct (WD), appears at the level of the future hind limbs. Then the ureteric bud invades a condensation of the intermediate mesoderm, called metanephric mesenchyme, and is induced by metanephric mesenchyme to branch from E11.5 onwards to develop to the renal collecting duct system [4], [5]. The primary stalk of the ureteric bud that connects the developing kidney first to the Wolffian duct and later to the bladder, develops to become the ureter. The most posterior Wolffian duct segment is called the common nephric duct (CND), which connects ureteric bud to urogenital sinus, the later bladder [5], [6]. In later developmental stages, the CND undergoes apoptosis to let the ureter join urogenital sinus directly [7]. The ureter budding site along the Wolffian duct as well as the appropriate CND absorption process are important to the final position of ureterovesical junction and distal ureter maturation.

During ureter development, the epithelial cells differentiate into the urothelium, while a layer of smooth muscle cells are differentiated from the condensed mesenchymal cells around the ureteric epithelium, and mediate peristalsis, conducting urine from the renal pelvis to bladder. In later stage, another kind of mesenchymal cells is differentiated between smooth muscle layer and epithelium in ureter, called subepithelial ureteral mesenchymal cells. Recent report revealed that Shh from ureteric epithelium is required for differentiation of subepithelial ureteral mesenchymal cells. Deletion of *Shh* in urothelium causes absent of subepithelial ureteral mesenchymal cells. The mutant mice display congenital renal hypoplasia, hydronephrosis and hydroureter phenotype at birth [8].

BMP signaling pathway is essential for many development processes. During ureter development, *Bmp4* and *Bmp5* are expressed in ureteral mesenchymal cells, while Bmp7 is expressed in ureteric epithelium [9]. Gene targeting approaches have uncovered some of their important roles during ureter development. *Bmp*7 deficient mice display a dysplastic kidney and hydroureter phenotype [10], [11]. Mice heterozygous for a null mutation of *Bmp4* display abnormalities that mimic human congenital anomalies of the kidney and urinary tract (CAKUT), suggesting that *Bmp4* has important functions in the early development of urinary tract by inhibiting ectopic budding from WD or the ureter stalk [12]. At later stage, *Bmp4* is reported to have multiple biological functions in urinary system development. For instance Bmp4 can act on the metanephric mesenchyme, prevents cell death and promotes expansion and migration of mesenchymal cells [13]. *Fstl1* encodes a secreted extracellular glycoprotein that belongs to the BM/SPARC/osteonectin family, which contains an extracellular calcium-binding (EC) domain and a follistatin (FS)-like domain [14], [15]. In zebrafish, morpholino knockdown of *zFstl2* (the mouse *Fstl1* homolog) results in a ventralized body axis [16]. Recently, *Fstl1* is indicated to act as a BMP4 signaling antagonist in lung development [17]. Nevertheless, how Fstl1 affects BMP signaling or the function of *Fstl1* during ureter development remains unclear.

Clues about Fstl1 function in the developing urinary system is suggested by its dynamic expression in the nephric duct and the nascent nephron epithelia of the developing kidney [18]. In this study, we report that *Fstl1* knockout mice (*Fstl1^-/-^*) display a prominent hydroureter beginning at E16.0. Our histological and biochemical results suggest that Fstl1 has important roles in regulating ureteric epithelial cell proliferation, subepithelial ureteral mesenchymal cells differentiation and distal ureter maturation, mainly via regulating BMP and SHH signaling pathways.

## Materials and Methods

### Ethics Statement

This study was approved by the Institutional Animal Care and Use Committee of Model Animal Research Center, Nanjing University, in strict accordance with the Guide for the Care and Use of Laboratory Animals, China. The relevant approved animal protocol (MARC-AP#: XG28) is entitled “*Fstl1* antagonizes BMP signaling by regulating epithelial-mesenchymal interaction during ureter development”.

### Mouse Strains

*Fstl1* floxed mice (*Fstl1^flox/+^*) were generated by inserting two *loxP* sites into intron 1 and intron 2 respectively, followed by a neomycin cassette and a third *loxP* site in same orientation [19]. Heterozygous *Fstl1* knockout mice (*Fstl1* ^+/-^) were generated by crossing *Fstl1^flox/+^* mice with EIIa-Cre mice (B6.FVB-Tg(EIIa-cre)C5379Lmgd/J, 003724), in which Cre recombinase expression occurs prior to implantation in the uterine wall [20]. Intercross of *Fstl1* ^+/-^ mice produced null mutant mice (*Fstl1* ^-/-^) with exon 2 deletion. *Ptch-lacZ^+/–^* mice (STOCK *Ptch1^tm1Mps^*/J, 003081) were obtained from The Jackson Laboratory (Bar Harbor, ME). *Fstl1* knockout mice (*Fstl1^+/-^*) as well as *Fstl1* ^+/-^;*Ptch-lacZ* ^+/–^ mice were kept at 129;B6 mixed background. All mice were maintained in an AAALAC accredited SPF facility in Model Animal Research Center of Nanjing University.

### Histology and Immunohistology

For histological staining, urinary system was fixed in 4% paraformaldehyde, paraffin-embedded, sectioned (6µm), and stained with hematoxylin-eosin. Immunohistochemistry was performed on paraffin sections following a standard protocol with antibodies listed below [21].

For X-gal staining of urinary systems, the whole urinary tract was fixed for 30min in fixative buffer (0.05 M NaPO_4_ buffer, PH 7.3, 1.8% formaldehyde, 0.02% NP-40, 2 mM MgCl_2_, 0.01% deoxycholate) on ice. After fixation, the urinary systems were washed 5 min in rinse buffer (0.05 M NaPO_4_ buffer, PH 7.3, 0.02% NP-40, 2 mM MgCl_2_) for three times and stained by immersion in X-gal staining solution (0.05 M NaPO_4_ buffer, PH 7.3, 2 mM MgCl_2_, 5 mM potassium ferrocyanide, 5 mM potassium ferricyanide, 0.1% X-gal) overnight at 37°C in the dark. The X-gal-stained urinary systems were rinsed with PBS, and post-fixed with 4% paraformaldehyde in PBS at 4°C. Then the samples were paraffin embedded and sectioned (10 µm). The sections were counterstained with nuclear fast red.

### Antibodies

Antibodies and reagents used are as follows: goat anti-Fstl1 (R&D system, AF1738), goat anti-Smad1 (R&D system, AF2039), rabbit anti-phospho-Smad1/5/8 (Cell Signaling Technology, 9511), rabbit anti-Pax2 (Zymed, 71–6000), DAPI (Sigma-Aldrich, D9542), rabbit anti-pan-Cytokeratin (Santa Cruz Biotechnology, SC-15367), mouse anti-α-SMA (Neomarkers, MS-113-P1), goat anti-SM22 α (Abcam, ab10135), mouse anti-smMHC (Abcam, ab53219), mouse anti-phospho-AKT (Ser^473^) (Cell Signaling Technology, 4051), rabbit anti-AKT (Cell Signaling Technology, 9272), mouse anti-β-actin (Sigma, A5441), mouse anti-GAPDH (Santa Cruz Biotechnology, SC-32233), mouse anti-c-Myc (Santa Cruz Biotechnology, SC-40), mouse anti-HA (Sigma-Aldrich, H3663), FITC-conjugated mouse (Sigma-Aldrich, F5262) and Cy5-conjugated rabbit (Biomeda Corp., SJ29004) secondary antibodies and DAB (Maixin, KIT-9710), Goat anti-mouse IgG (Fc) (Pierce, 31439), Rabbit anti-Goat IgG (Sigma-Aldrich, A5420), and Goat anti-Rabbit IgG (Sigma-Aldrich, A9169).

### Dye Injection

To visualize the urinary tract lumen, Bromophenol blue was injected into the pelvic region of the kidneys using a three dimensional manipulator (MN-153, NARISHIGE).

### Analysis of Peristalsis

E18.5 kidneys and associated ureters were dissected from wild-type and *Fstl1^-/-^* embryos and were incubated in an *in vitro* culture chamber attached to an Olympus X71 inverted microscope. Ureter movements were observed and captured by digital camera of the microscope apparatus.

### Proliferation and Apoptosis Analysis

To determine proliferative activity of the developing ureter, Timed-mated pregnant females were intraperitoneal injected with 10µl/g body weight BrdU (5mg/ml) (Sigma-Aldrich). E15.5 urinary systems were harvested 1 hour after injection, and E16.5 urinary systems were collected 2 hours after injection. For E15.5 embryos, a total of over 20 ureter sections from three embryos of each genotype were collected for quantification. For E16.5 embryos, a total of 20 ureter sections from the distal segment of ureter of each genotype were collected for quantification. Proliferative activity was examined after treatment with Fstl1 in culture: Wild-type E15.0 urinary system (kidney, ureter and bladder) was dissected and positioned on top of a culture plate insert (0.4 µm pore size, Millipore Corporation, Bedford, MA01730, USA) within an individual well of a 24-well tissue culture plate and cultured in mock or Fstl1-containing conditioned media for 16 hours, with 10 µM BrdU added during the last 4 hours of treatment [22]. A total of 7 sections from 3 pairs of cultured ureters were used for quantification. The BrdU-labeling index was defined as the number of BrdU-positive nuclei relative to total number of nuclei, which were counterstained with hematoxylin.

Apoptotic cells were detected using TUNEL assay that performed on 6µm paraffin sections using DeadEnd^TM^ Fluorometric TUNEL System (Promega, G3250). For both E15.5 and E16.5 embryos, a total of 6 ureter sections from two embryos of each genotype were analyzed. For E12.5 embryos, a total of 6 sections containing CND region from two embryos of each genotype were analyzed.

### In Situ Hybridization

*In situ* hybridization was performed as previously described [23]. A 619 bp 3’ UTR fragment of *Fstl1* cDNA was subcloned into the pBluescript II KS (-) (Stratagene) plasmid, which was used to generate an *in situ* hybridization probe (PCR primers are listed in Table S1). *Pax3* probe was kindly provided by Dr. Yingzi Yang from NIH as a gift. *Pax2* probe was provided by Dr. Yeguang Cheng (Tsinghua University, Beijing, China) as a gift. 3 embryos of each stage were used for *Fstl1* expression examination. 4 embryos of each genotype were used for whole mount *in situ* hybridization using *Pax2* and *Pax3* probes.

### Quantitative Real-Time PCR

The ureters were dissected and separated from the rest of the kidney. Two pairs of ureter were pooled into one sample. Over 4 groups of each genotype were used for real-time PCR for each gene. Total RNA from E16.5 ureter samples was isolated using the Qiagen RNeasy Mini kit (Qiagen). First strand cDNA was synthesized using AMV reverse transcriptase (Takara). Primers targeting specific transcripts were designed for real-time RT-PCR (SYBR). *β-actin* was used as internal control in each reaction. Quantitative real-time PCR was performed using an ABI PRISM 7700 (ABI) with conditions recommended by the manufacturer. Each reaction was performed triplicate. The quantity of each experimental sample is first determined using a standard curve based on their Ct values and then expressed relative to the internal control. The primers used to amplify each gene are listed in Table S1.

### Cell Culture and Transfection

HEK-293 (CRL-1573^TM^, ATCC) cells were routinely cultured in DMEM supplemented with 10% FBS. Fstl1 coding sequence was subcloned into pcDNA3.1 (Invitrogen) vectors to express Fstl1 with (pcDNA3.1- Myc-Fstl1) or without Myc-tag (pcDNA3.1- Fstl1). HA tagged ALK3, ALK5 and ALK6 plasmids were provided by Dr. Yeguang Cheng (Tsinghua University, Beijing, China) as gifts.

To prepare the conditioned media, pcDNA3.1-Fstl1 or pcDNA3.1 vector (Mock) were transfected into cells using Lipofectamine 2000 (Invitrogen) following the manufacturer’s instructions. 24 hours after transfection, supernatant medium was collected as conditioned media for following treatment. The cells subject to conditioned media treatment were plated at 3×10^5^ per well in 12 well plate, and starved for 4 hours before medium changed. Cells were treated with the conditioned media in addition of BMP4 (20 ng/ml) (PeproTech, Rocky Hill, NJ) for 30 min.

### Immunoprecipitation and Western Blot

For immunoprecipitation assay, HEK293 and COS7 cells were co-transfected with pcDNA3.1-Myc-Fstl1 and Bmp receptors expression plasmids HA-ALK3, ALK6 or ALK5. After transfection, the cells were cultured for 48 hours. Whole cell lysate was incubated with anti-c-Myc antibody, and rotated at 4°C for 6 hours. Then Protein A/G Agarose beads (Biyuntian Bio) were added and incubated overnight at 4°C with rotation.

Protein extracts (50µg/lane) were separated on discontinuous 10% SDS-PAGE gel followed by transfer to PVDF membrane (Amersham Biosciences), 100V for 1 hr. The transferred membrane was blocked in 5% skim milk in TTBS (0.1% Tween-20 in TBS) buffer for 1hr at RT, then immunoblotted with primary antibody overnight at 4°C. After four washes with TTBS for 10 min each, the membrane was incubated with peroxidase-coupled secondary antibody for 1 hr at RT. After three washes in TBS for 10 min each, the immuno-reactive bands were visualized with a chemiluminescent substrate for peroxidase (Super Signal West Pico substrate, Pierce). For ureter samples, ureters were dissected and separated from the rest of the kidney. 10 pairs of ureters from each genotype were pooled together as one sample. The experiments from both E15.5 and E16.5 ureters were duplicated. The results on E18.5 kidney samples were confirmed with more than 4 litters of embryos.

### Statistics

All results were presented as mean ± SEM. All statistical analyses were done using GraphPad Prism5 software. Two-tailed Student’s *t* tests were used for comparisons between two groups. *, *p*<0.05; **, *p*<0.01; ***, *p*<0.001. *p*<0.05 was considered significant.

## Results

### Fstl1 is Expressed in Developing Ureter

**Figure 1:**
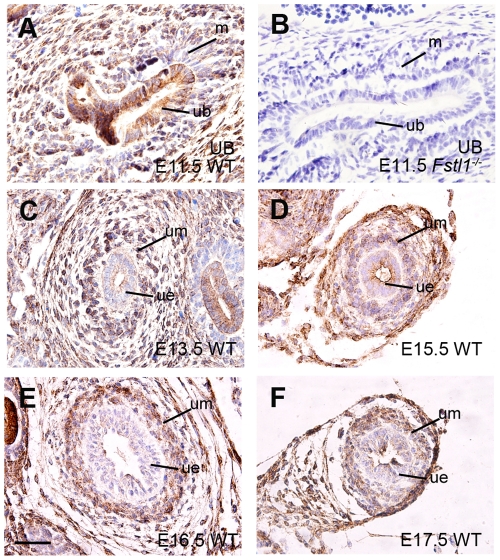
Fstl1 protein expression in developing murine ureter. Fstl1 immunohistochemistry analysis in sagittal sections of WT (A) and *Fstl1* ^-/-^ ureteric bud (B, negative control for antibody) at E11.5 and transverse sections of ureters at E13.5 (C), E15.5 (D), E16.5 (E) and E17.5 (F). Note that Fstl1 immunostaining was observed in the mesenchyme (um) as well as the ureteric epithelium (ue) from E15.5 to E17.5 (C-F). Scale bars: 40 µm. ub: ureter bud; m: metanephric mesenchyme; um: ureteral mesenchyme; ue: ureteric epithelium.

We examined *Fstl1* expression in the developing ureter by immunohistochemistry (IHC) at E11.5 (Figure. 1A), E13.5 (Figure. 1C), E15.5 (Figure. 1D), E16.5 (Figure. 1E), and E17.5 (Figure. 1F). IHC result of E11.5 *Fstl1* null embryo was presented as negative control (Figure. 1B). In developing ureter, Fstl1 protein was detected in both epithelial cells of ureteric bud and the surrounding mesenchymal cells (Figure. 1A) as early as E11.5. In later stages, Fstl1 was detected in the mesenchyme (inner and outer layers) and epithelium of the developing ureter at E13.5 (Figure. 1C), E15.5 (Figure. 1D), E16.5 (Figure. 1E), and E17.5 (Figure. 1F).

Since Fstl1 is a secreted glycoprotein, *Fstl1* mRNA expression was also examined by *in situ* hybridization. *Fstl1* mRNA was mainly produced in ureteral mesenchymal cells at E13.5 (Figure. S1A, B) and E15.5 (Figure. S1C, D). The difference of expression pattern of Fstl1 protein and RNA in ureter indicated that the Fstl1 protein found in the ureteric epithelium might be derived from the ureteral mesenchymal cells by diffusion.

### Fstl1^-/-^ Embryos Developed Congenital Hydroureter and Hydronephrosis

To study the function of *Fstl1* in development and pathophysiology, *Fstl1* conventional knockout allele was generated by crossing heterozygous conditional knockout mice (*Fstl1^flox/+^*) [19] with EIIa-Cre mice (B6.FVB-Tg (EIIa-cre) C5379Lmgd/J, 003724) [20].

**Figure 2:**
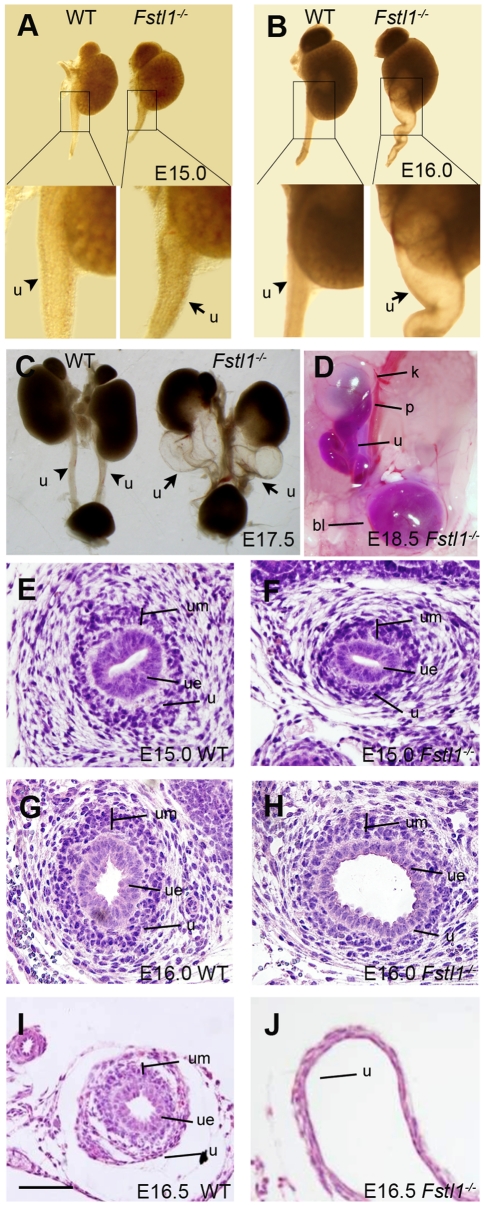
Histological analysis of the *Fstl1^-/-^* urinary system. (A-C) Kidneys and ureters from WT and *Fstl1^-/-^* embryos at stages of E15.0 (A), E16.0 (B) and E17.5 (C). Arrows indicate mutant ureters, and arrowheads indicate wild-type ureters. (D) Dye injection experiments detected no complete physical obstruction of the *Fstl1* ^-/-^ ureter at E18.5. (E-J) H&E staining on transverse sections of WT (E, G, I) and *Fstl1* ^-/-^ (F, H, J) ureters at E15.0 (E, F), E16.0 (G, H) and E16.5 (I, J). (E, F) No obvious change was detected at E15.0 in *Fstl1* ^-/-^ ureter. (G, H) *Fstl1* ^-/-^ ureter became dilated from E16.0. (I, J) There are prominent changes in the histological structure of the *Fstl1* ^-/-^ ureter compared to the WT ureter at E16.5. Scale bar: (E-J) 40 µm. k, kidney; p, pelvis; u, ureter; bl, bladder; um: ureteral mesenchyme; ue: ureteric epithelium.

*Fstl1* ^-/-^ mice died shortly after birth due to severe lung developmental defects [17], [24]. We found *Fstl1 *mutant embryos also displayed profound hydroureter and hydronephrosis at birth. In order to define the onset and progression of urinary tract malformations in *Fstl1* ^-/-^ embryos, we analyzed urinary systems of wild-type and *Fstl1* ^-/-^ embryos from E15.0 to E17.5 (Figure. 2A, B, C). Morphologically, obvious ureter dilatation was observed from E16.0 (Figure. 2B) in *Fstl1* ^-/-^ ureters, but was not detected at as early as E15.0 (Figure. 2A). The dilation was more severe in the proximal region than distal part of the ureter. At E17.5, mutant urinary tracts displayed prominent hydroureter phenotype. The mutant ureters were dilated, convoluted, fluid-filled and increased in length (Figure. 2C). All these abnormalities occurred bilaterally and were fully penetrant in both male and female null mutants.

At the histological level, HE staining did not show any obvious differences between wild-type and *Fstl1* ^-/-^ ureters in the proximal segment at E15.0 (Figure. 2E, F). At E16.0, the ureter became dilated from the proximal fragment (Figure. 2G, H). At E16.5, the urothelium of wild-type embryo was multilayered and surrounded by multiple layers of mesenchymal cells (Figure. 2I). In contrast, the *Fstl1* ^-/-^ ureter was enlarged with thinner layers of urothelium and mesenchyme (Figure. 2J).

Kidney was also examined. Since *Fstl1* was highly expressed in collecting duct in kidney at E17.5 [18], Pax2 expression was examined as a colleting duct marker. From E15.5 to E17.5, Pax2 expression level in the collecting duct of mutant kidneys was normal (Figure. S2). However, from these histological results, we found that *Fstl1* mutant kidney was reduced in size and displayed the dilatation of the pelvis and atrophy of papilla at E17.5, suggesting *Fstl1* ^-/-^ kidneys exhibited hydronephrosis at this stage (Figure. S2E, F). Obvious enlargement of renal pelvis was not observed in *Fstl1* embryo at E15.5 (Figure. S2A, B) and E16.5 (Figure. S2C, D), therefore we speculate that hydronephrosis was possibly a secondary consequence of hydroureter.

Physical obstruction is a potential cause of ureter dilation during urinary system development. To determine whether there was complete physical obstruction along the urinary tract in *Fstl1* mutant embryo, we injected dye directly into the pelvic region of E18.5 *Fstl1* ^-/-^ kidneys. The purple dye could flow through the urinary path to the bladder (Figure. 2D). This result indicates that the hydroureter phenotype wasn’t caused by a complete obstruction.

### Defects of Distal Ureter and Ureterovesical Orifice in Fstl1^-/-^ Embryos

**Figure 3:**
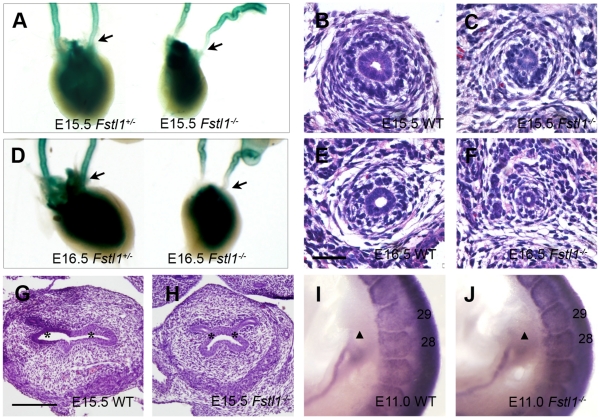
Defects of UV orifice and distal ureter in *Fstl1^-/-^* embryo. (A-F) *Fstl1* ^+/-^ mice were crossed to *Ptch-lacZ* ^+/-^ mice. *Fstl1* ^+/-^_;_ *Ptch-lacZ* ^+/-^ and *Fstl1* ^-/-^_;_ *Ptch-lacZ* ^+/-^ ureters were stained for β-galactosidase (blue) at E15.5 (A) and E16.5 (D). The *Fstl1^-/-^* and wild-type ureters were paraffin sectioned and stained with hematoxylin-eosin at E15.5 (distal segment) (B, C), E16.5 (distal segment) (E, F). Note that the *Fstl1* ^-/-^ ureter (A, D, C, F) is narrower than wild-type ureter (A, D, B, E) at both E15.5 and E16.5. The arrows indicated that lacZ staining was reduced in distal part of ureters in *Fstl1* ^-/-^ embryos compared to wild-type embryos. (G, H) Histological analysis of the ureterovesicle orifice at E15.5. Note that the distance between the left and right orifices (asterisk) is shorter in the *Fstl1* ^-/-^ embryo (H), compared with the wild-type embryo (G) at E15.5. (I, J) At E11.0, *Pax2* and *Pax3* whole mount *in situ* hybridizations were performed for wild-type (*n* =  4) (I), *Fstl1^-/-^* (*n* =  4) (J). The somites, which were labeled by the *Pax3 in situ* probe, in the caudal region of the embryo were numbered. The initial site of ureteric bud, which was labeled by *Pax2* and indicated by a triangle, in both the wild-type (I) and *Fstl1^-/-^* (J) embryos aligned with the 28^th^ somite level. Scale bar: (B-F) 40µm, (G, H) 100µm.

Ureterovesical junction defects were observed in many hydroureter mouse models [25], [26] as well as human patients with CAKUT [1]. Previous report also revealed that Bmp4 heterozygote mutant embryos display ureterovesical junction defects with high penetrance [12]. *Fstl1* is suggested to be a BMP signaling antagonist [17]. Therefore, we examined the ureterovesical orifice as well as the distal ureter of *Fstl1^-/-^* embryos. We found that the very distal segment of *Fstl1^-/-^* ureters was extremely narrow (Figure. 3A, D, right, C, F) compared with wild-type (Figure. 3A, D, left, B, E) ureter at E15.5 (Figure. 3A, B, C) and E16.5 (Figure. 3D, E, F). Histological analysis also revealed that distance between the two ureteral orifices is significantly shorter in *Fstl1^-/-^* embryos (Figure. 3H, Figure. S3B, D, I) than in wild-type (Figure. 3G, Figure. S3A, C, I) embryos at E15.5 (Figure. 3G, H, Figure. S3A, B, I) and E16.5 (Figure. S3C, D) which was similar to the ureterovesical junction defects in *Bmp4^+/-^* embryo [12]. These results suggested that *Fstl1* deficiency caused developmental defects in the distal part of ureter and ureterovesical junction.

During the development of urinary tract, ureteric bud first binds to the Wolffian duct, migrates to cloaca, and finally joins bladder. The abnormality of ureterovesical orifice could be caused by ectopic initial ureteric budding site or by defects of subsequent processes, such as the absorption of common nephric duct (CND) [7], [12], [25], [27], [28]. Therefore, we examined ureterovesical junction in earlier developmental stages. At E11.0, we investigated whether aberrant ureteral budding occurred. Wild-type (*n* = 4) and *Fstl1* ^-/-^ (*n* = 4) embryos were subjected to *in situ* hybridization using *Pax2* and *Pax3* antisense probes. *Pax2* is expressed in the epithelial ureteric bud in the urinary system [29], while *Pax3* is expressed in many tissues, including the somites [30]. Then we analyzed the embryos after *in situ* hybridization of both probes, and we found that both in wild-type (Figure. 3I) and *Fstl1* ^-/-^ (Figure. 3J) embryos, the position of the initial ureteric budding site aligned with the 28^th^ somite. These observations demonstrated that position for initial ureteric bud outgrowth along the Wolffian duct was not affected by *Fstl1* deficiency.

At E12.5, when the ureter still binds the WD, the CND is actively absorbed into the cloaca by apoptosis [7]. We thus examined the length and apoptotic level of the CND in *Fstl1* ^-/-^ embryos. At this stage, the length of CND was similar in both wild-type (Figure. S3E) and *Fstl1* ^-/-^ (Figure. S3F) embryos. The apoptosis of CND was detected by TUNEL assay. TUNEL positive nuclei were highly localized in the epithelium of the CND (Figure. S3G, H). No obvious difference of CND apoptosis was detected between wild-type (Figure. S3G, J) and *Fstl1* ^-/-^ (Figure. S3H, J) embryos. These results suggested that the defects of ureterovesical junction and distal ureter in *Fstl1* mutant embryo are not caused by ectopic ureteric budding site or inappropriate regression of CND.

### Inactivation of Fstl1 Results in Defective Ureteric Epithelial Cell Proliferation and Differentiation

The hydroureter is often associated with cell proliferation and/or apoptosis defects during ureter development [8], [13], [31], [32], [33], [34]. Beside the dilation of ureter at proximal part, we also found the distal ureter of *Fstl1* mutant embryo is significantly narrower than that of wild-type embryo. Therefore, we examined proliferation and apoptosis in *Fstl1* ^-/-^ embryonic ureters. Very few cells in wild-type and *Fstl1^-/-^* ureters underwent apoptosis at E15.5 as determined by the TUNEL assay. There was no significant difference in ureteric epithelium and ureteral mesenchyme between wild-type and mutant embryos (Figure. S4A, B). Similar results were obtained in E16.5 ureters (Figure. S4C, D).

**Figure 4:**
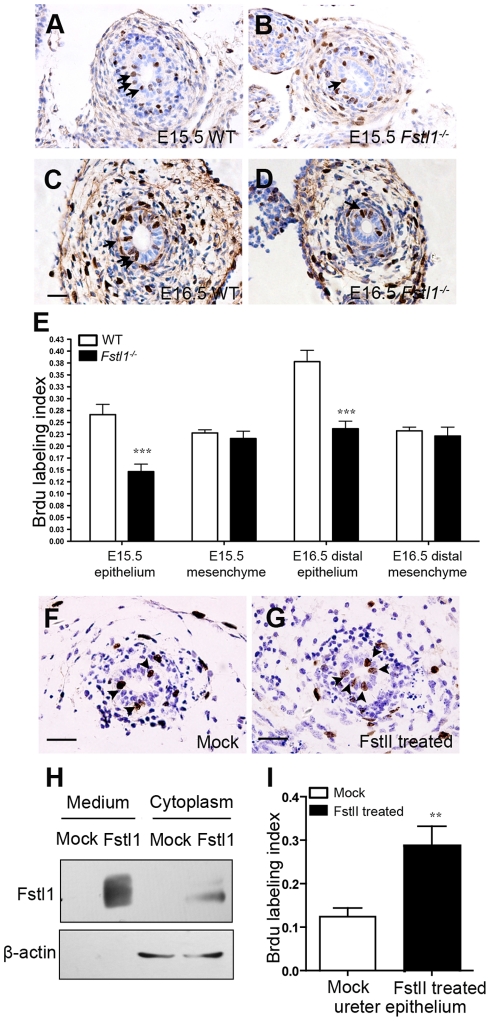
Proliferation defects in *Fstl1^-/-^* ureteric epithelial cells. (A-D) Analysis of cell proliferation on transverse sections of wild-type (A, C) and *Fstl1^-/-^* (B, D) ureters by BrdU incorporation at E15.5 (A, B) and E16.5 (distal segment) (C, D). Arrows point to representative proliferating cells. (E) Quantification of cell proliferation by BrdU labeling index. In E15.5 ureter, *p*<0.001 for epithelial layer (*n* = 20) and p = 0.49 for mesenchymal inner layer (*n* = 20). In distal segment of E16.5 ureter, *p*<0.001 for epithelial layer (*n* = 20) and p = 0.59 for mesenchymal inner layer (*n* = 20). (F, G) BrdU incorporation analysis of epithelial cell proliferation on transverse section of cultured E15.0 ureters treated with conditioned media transfected either with pcDNA3.1 vector (Mock) (F) or an Fstl1 overexpress plasmid (G). Arrows point to representative proliferating cells. (H) HEK293 cells were transfected with a plasmid expressing mouse Fstl1 protein or pcDNA3.1 (mock). Western blot analysis using an anti-Fstl1 antibody indicated that Fstl1 was overexpressed in both the cultured media and cell pellet. (I) Quantification of cell proliferation by BrdU labeling index in the cultured E15.0 ureteric epithelium. *p*<0.005 (*n*  = 7). Scale bar: (A-D, F-G) 20µm.

We examined proliferation of ureteric epithelium and ureteral mesenchyme by BrdU incorporation in E15.5 wild-type and *Fstl1^-/-^* ureters (Figure. 4A, B, E). There was no change in the rate of mesenchymal cell proliferation (Figure. 4E). On the contrary, proliferation of *Fstl1^-/-^* ureteric epithelium was significantly reduced at E15.5 (Figure. 4E). At E16.5, the proximal segment of *Fstl1^-/-^* ureter was already dramatically dilated. Therefore, we analyzed the proliferation rates of distal segment of ureter, which was narrower in *Fstl1^-/-^* embryo compare to wild-type. Similar with E15.5, BrdU incorporation indicated that the proliferation of ureteric epithelial cell was significantly decreased in distal segment of *Fstl1^-/-^* ureter (Figure. 4C, D, E), and the ureteral mesenchymal cell proliferation was not affected by *Fstl1* deficiency (Figure. 4C, D, E).

To further confirm the effect of Fstl1 on ureteric epithelium proliferation, we cultured wild-type E15.0 ureters with conditioned media from cells transfected with pcDNA3.1 vector (Mock) (Figure. 4F) or Fstl1 expression plasmid (Figure. 4G). The presence of Fstl1 in conditioned media was confirmed by western blot (Figure. 4H). Proliferation of epithelial cells was significantly increased in wild-type ureters treated with Fstl1-containing media compared to control media, as quantified by BrdU incorporation (Figure. 4I). Our results indicate that Fstl1 is required for maintaining normal ureteric epithelial cell proliferation during development.

To determine whether urothelium differentiation was affected in *Fstl1* deficient mice, we examined the expression of *Upk3a,* a urothelium differentiation marker [35], [36], in E15.5 and E16.5 ureters. Expression of *Upk3a* was down-regulated in *Fstl1^-/-^* ureters compared to wild-type at E15.5 and E16.5 as determined by real-time PCR (Figure. S5), suggesting that urothelium differentiation is also impaired in the *Fstl1^-/-^* ureter.

### Subepithelial Ureteral Mesenchymal Cells were Absent in Fstl1^-/-^ Ureter

Previous studies have suggested that the hydroureter may result from defects in ureteral mesenchymal cell differentiation [8], [31]. So we examined the expression of mesenchymal cell markers in the *Fstl1* ^-/-^ ureter. At E15.5, the expression level of smooth muscle differentiation markers α-SMA (Figure. S6A, B), SM22α (Figure. S6C, D), and smMHC (Figure. S6E, F) were indistinguishable between wild-type (Figure. S6A, C, E) and *Fstl1* ^-/-^ ureters (Figure. S6B, D, F). Western blot assay also confirmed that the expression level of these three markers was not changed in *Fstl1* ^-/-^ ureter at E16.5 (Figure. S6G).

To further examine the contractile function of ureter smooth muscle, E18.5 (Movie S1, S2) kidneys and associated ureters were isolated and cultured. Ureter movements were observed. We found that even in late stages that the morphology of mutant ureters dramatically changed, contraction of the smooth muscle seemed to be normal in *Fstl1^-/-^* embryos (Movie S2) compared with wild-type embryos (Movie S1).

**Figure 5:**
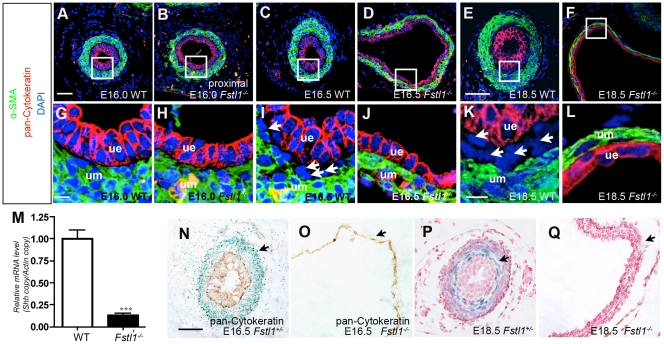
Subepithelial ureteral mesenchymal cells were absent in the *Fstl1* ^-/-^ ureter. (A-L) Immunofluorescence staining of α-SMA (green), pan-Cytokeratin (red), and DAPI (blue) of transverse sections from WT ureters at E16.0 (A, G), E16.5 (C, I) and E18.5 (E, K), and *Fstl1* ^-/-^ ureters at E16.0 (B, H), E16.5 (D, J) and E18.5 (F, L). (G-L) Enlarged views of the boxed area in (A-F). The arrows indicate subepithelial ureteral mesenchymal cells which are negative for both markers. (M) Quantitative real-time PCR of Shh (*n* = 5, *p* = 0.0008) from E16.5 ureters. (N-Q) *Fstl1* ^+/-^ mice were crossed to *Ptch-lacZ* ^+/-^ mice. *Fstl1* ^+/-^_;_ *Ptch-lacZ* ^+/-^ and *Fstl1* ^-/-^_;_ *Ptch-lacZ* ^+/-^ ureters were stained for β- galactosidase (blue) and pan-Cytokeratin (brown) at E16.5 (N, O); β-gal (blue) and nuclear fast red (red) at E18.5 (P, Q). Note that the lacZ staining was reduced in *Fstl1* ^-/-^ ureters (O, Q) compared to *Fstl1* ^+/-^ ureters (N, P). Scale, bar: (A-F) 50µm, and (G-L) 10µm, (N-Q) 40µm. um: ureteral mesenchyme; ue: ureteric epithelium.

A layer of α-SMA-negative mesenchymal cells was differentiated between the ureteric epithelium and the multilayer α-SMA-positive mesenchymal cells in later developmental stages [8]. These cells, referred to as the subepithelial ureteral mesenchymal cells, are also absent in *Shh* and *Dlgh1* mutants displaying hydroureter phenotype [8], [37]. We traced differentiation of these cells in both wild-type and *Fstl1* ^-/-^ ureters from E15.5 to E18.5, by double staining of epithelial cell marker pan-cytokeratin and smooth muscle cell marker α-SMA. At E15.5 and E16.0, we could not detect that type of cells negative for both of these markers in either wild-type (Figure. S7A, C and Figure. 5A, G) or *Fstl1* mutant ureter (Figure. S7B, D and Figure. 5B, H). But from E16.5 on, subepithelial ureteral mesenchymal cells developed in wild-type ureter (Figure. 5C, I, arrows) and became a thicker cell layer at E18.5 (Figure. 5E, K, arrows). However, this layer of cells was always missing in the *Fstl1* ^-/-^ ureter at E16.5 (Figure. 5D, J) and E18.5 (Figure. 5F, L). We didn’t detect any cell that is negative for both markers in dilated *Fstl1^-/-^* ureters.

SHH signaling pathway is crucial for subepithelial ureteral mesenchymal cells differentiation and ureter development [8]. Therefore, we analyzed *Shh* expression in the developing ureter at E16.5. We found that *Shh* was down-regulated in the *Fstl1^-/-^* ureter as determined by real-time PCR (Figure. 5M). Ptch is the receptor of Shh as well as one of the downstream targets of SHH signal [38]. Using *Ptch-lacZ* ^+/–^ mice, in which lacZ is knocked-in to the *Ptch* locus, subepithelial ureteral mesenchymal cells can be exclusively labeled by X-gal staining at E18.5 [8], [38]. Therefore, we used this mouse strain to examine SHH signaling in *Fstl1* mutants. X-gal staining at E16.5 and E18.5 was performed. At E16.5, Ptch-lacZ was expressed in mesenchymal cells in wild-type ureters (Figure. 5N). X-gal staining signal was weaker in the *Fstl1* ^-/-^;*Ptch-lacZ* ^+/–^ ureters (Figure. 5O) than in the *Fstl1* ^+/+^;*Ptch-lacZ* ^+/–^ ureters (Figure. 5N). In E18.5 embryos, X-gal staining signal in *Fstl1* ^+/+^;*Ptch-lacZ* ^+/–^ ureters was strong in subepithelial ureteral mesenchymal cells (Figure. 5P, arrow), whereas no signal was detected in *Fstl1* ^-/-^;*Ptch-lacZ* ^+/–^ littermates (Figure. 5Q, arrow). These results confirm that subepithelial ureteral mesenchymal cells are absent, and that SHH signaling is down-regulated in *Fstl1^-/-^* ureters.

### Fstl1 Deficiency Led to up-regulation of BMP Signaling in Developing Ureter and Kidney

**Figure 6:**
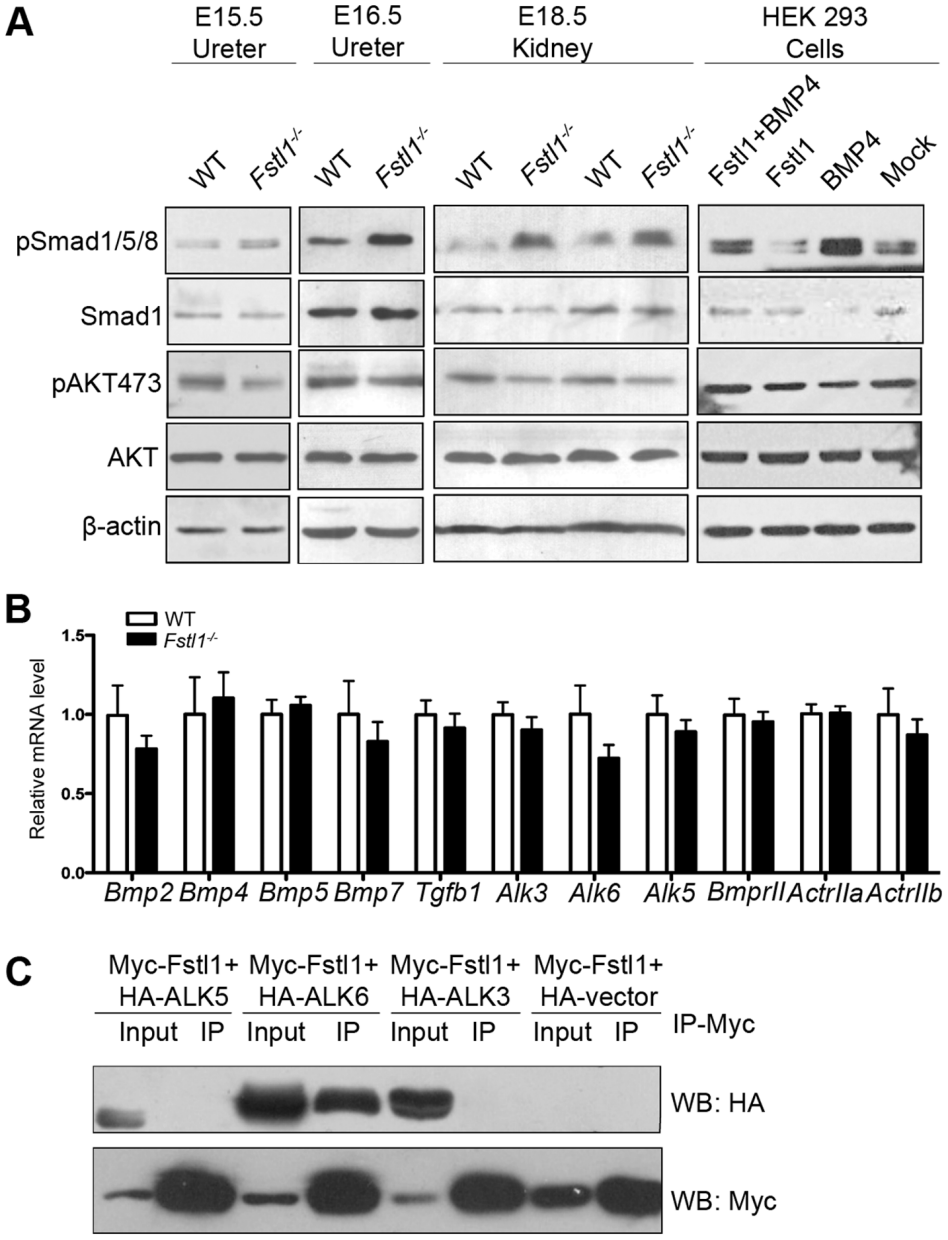
Up-regulation of BMP signaling in the *Fstl1^-/-^* ureter and kidney. (A) Western blots of pSmad1/5/8, Smad1, pAKT (Ser^473^), AKT, and β-actin from E15.5 (left panel) and E16.5 (second panel) ureter protein, E18.5 kidney protein (third panels), and HEK293 cells treated with the conditioned media containing BMP4 (20 ng/ml) transfected either with the Fstl1 or pcDNA3.1 vector (Mock) for 30 min (right panels). (B) Quantitative real-time PCR of *Bmp2* (*n* = 5, *p* = 0.35), *Bmp4* (*n* = 4, *p* = 0.73), *Bmp5* (*n* = 5, *p* = 0.63), *Bmp7* (*n* = 5, *p* = 0.50), TGF-β1 (*n* = 5, *p* = 0.51), *Alk3* (*n* = 6, *p* = 0.40), *Alk6* (*n* = 5, *p* = 0.21), *Alk5* (*n* = 4, *p* = 0.47), *BmprII* (*n* = 6, *p* = 0.73), *ActrIIa* (*n* = 5, *p* = 0.97), and *ActrIIb* (*n* = 5, *p* = 0.73) from E16.5 ureters. (C) Co-immunoprecipitation of Myc-Fstl1 and HA-tagged BMP/TGF-β receptors in HEK293 cells. Myc-Fstl1 can be immunoprecipitated with the anti-c-Myc antibody. Note that HA-ALK6 was co-immunoprecipitated by the anti-c-Myc and detected by the anti-HA antibody (lane 4). Scale bar: 40µm.

Previous reports have suggested a correlation between Fstl1 and BMP signaling [16], [17], [39], and that BMP signaling is important for urinary system development [2]. We examined the effect of *Fstl1* deficiency on BMP signaling in the urinary system. Smad1/5/8 phosphorylation levels in the ureter and kidney were analyzed by western blot (Figure. 6A, first, second, and third panels). There was an increase of Smad1/5/8 activities in *Fstl1^-/-^* ureter at E15.5 (Figure. 6A, left panels), and E16.5 (Figure. 6A, second panels) compared to wild-type ureter. The increased Smad1/5/8 phosphorylation level was also detected in *Fstl1^-/-^* kidneys (Figure. 6A, third panels) at E18.5. Although the pattern of Smad1/5/8 phosphorylation didn’t change, the elevation of Smad1/5/8 phosphorylation level was confirmed by immunostaining of wild-type (Figure. S8A, C, E) and *Fstl1^-/-^* ureters (Figure. S8B, D, F) at E15.5 (Figure. S8A, B), 16.5 (Figure. S8C, D) and E18.5 (Figure. S8E, F).

TGF-β signaling was determined by western blots of phosphorylated Smad2. Smad2 activity was not affected by the *Fstl1* deficiency in E18.5 kidney or E15.5 ureter (Figure. S9).

Previous reports indicated that Fstl1 plays important roles in the cardiovascular system via AKT signal pathway [40], [41], so we also examined the effect of *Fstl1* deficiency on AKT signal. Phosphorylation level of AKT at Ser^473^ decreased in *Fstl1^-/-^* kidneys at E18.5 (Figure. 6A, third panels). But the AKT activity was not affected by Fstl1 deficiency in E15.5 (Figure. 6A, left panels) and E16.5 (Figure. 6A, second panels) ureter.

Since we detected an increased BMP signal in *Fstl1^-/-^* ureter, we then examined the levels of multiple BMP/TGF-β signal ligands and receptors, including *Bmp2*, *Bmp4*, *Bmp5*, *Bmp7*, *Tgf-β1*, *Alk3*, *Alk6*, *Alk5*, *BmprII*, *ActrIIa*, and *ActrIIb*. The expression level of these BMP/TGF-β signal ligands and receptors were not altered in *Fstl1^-/-^* ureter at E16.5 as determined by real-time PCR (Figure. 6B). These results are consistent with previous speculation that Fstl1 acted as a BMP antagonist [16], [17], [39].

To further confirm the function of Fstl1 on BMP signaling, we examined the effect of Fstl1 in HEK293 cells using Fstl1-containing conditioned media. Serum-starved HEK293 cells were treated with Fstl1-containing conditioned media and 20 ng/ml recombinant BMP4. Following BMP4 stimulation, BMP signal was activated. Smad1/5/8 phosphorylation level was lower in cells treated with Fstl1-containing conditioned media compared to the control conditioned media (Figure. 6A, right penals), suggesting that Fstl1 can antagonize BMP4-induced Smad1/5/8 activation in HEK293 cells. By performing a similar *in vitro* assay in HEK293 cells, we found that Fstl1 also had mild inhibitory effects on BMP2 (10 ng/ml) stimulation (Figure. S10A, lane 3, 5).

We then sought to identify potential ligand or receptor that physically interacts with Fstl1. Since BMP ligands and Fstl1 are both secreted proteins, we focused on identifying BMP receptors. We performed co-immunoprecipitations (co-IP) using Myc-tagged Fstl1 and HA-tagged BMP/TGF-β receptors, including ALK3, ALK5, and ALK6 in HEK293 cells. Using anti-HA antibody following IP from whole cell lysates with an anti-c-Myc antibody, we found that HA-ALK6 could be detected in IP protein from HEK293 cells (Figure. 6C). To confirm this result, we also performed same co-IP assay in COS7 cells, and found that both HA-ALK6 and HA-ALK3 could be detected in IP protein from COS7 cells (Figure. S10B). These *in vitro* results suggested that BMP type I receptors may be potential Fstl1 binding targets *in vivo* that mediates its antagonizing effect during ureter development.

## Discussion

**Figure 7:**
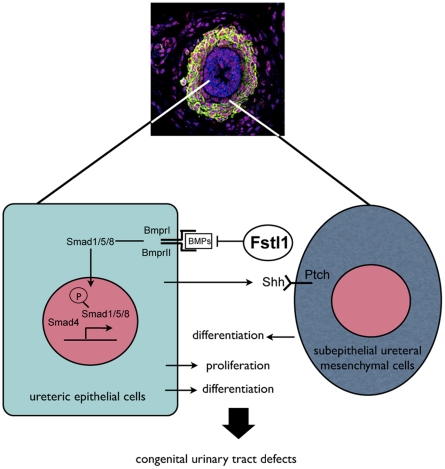
A model for Fstl1 function in urinary tract development. Fstl1 is mainly expressed in ureteral mesenchymal cells, secreted to the ureteric epithelial layer, and functions as an antagonist regulating BMP signaling. When Fstl1 is disrupted, ureteric epithelium and mesenchymal-epithelial interaction defects down-regulate SHH signaling, interfering with subepithelial mesenchymal cells differentiation and together leading to the hydroureter phenotype.

Ureter development is a complicated process involving organogenesis at least at two directions, the elongation of the ureter stalk and the differentiation of ureteric epithelial and mesenchymal layers. BMP and SHH signaling pathways have been reported to participate in regulation of these developmental processes. However, the precise control of these pathways remains obscure. In this study, we provided substantial evidences suggesting that Fstl1, a secreted protein, plays important roles during urinary tract development by antagonizing BMP signal (Figure. 7). Disruption of *Fstl1* causes multiple defects in developing urinary tract, including hydroureter arising from proximal segment as well as ureterovesical junction defects. Furthermore, *Fstl1* deficiency also results in down-regulated SHH signal, which in turn may affect subepithelial ureteral mesenchymal cells differentiation through mesenchymal-epithelial interaction (Figure. 7).

The function of FSTL1 on regulating BMP signaling may be mediated at both receptor and ligand level. For receptors, the key issue is that FSTL1 interacts with different targets depending on specific cell types. In our previous paper analyzing lung phenotype of *Fstl1* gene targeting mice, the Hep3B cell line was used mostly because of the endoderm tissue origin of lung epithelium [17]. For Hep3B cell line, FSTL1 can pull down BMPRII, but not BMPR1B (ALK6). In this study, we used HEK293 cell line which is derived from human embryonic kidney cells because we focused on ureter development. We found that only ALK6 can be co-precipitated with FSTL1 in HEK293. When we used another kidney derived cell line, COS7, both ALK6 and ALK3 can be co-precipitated. Nevertheless, Alk6 is specifically expressed in the ureteric epithelial cells in the urinary tract [12]. Consistent with the previous studies, our results suggest that Alk6 may be potential Fstl1 binding target *in vivo* that mediates its antagonizing effect during ureter development. It will be interesting, however, to elucidate the detailed mechanism how Fstl1 functions *in vivo* in different tissues in the future.

In addition, structure prediction and previous work in zebrafish, Xenopus and chick indicates that Fstl1 functions as a BMP antagonist similar to follistatin [16], [39], [42]. Because FSTL1 also binds to BMP(s) [17], [43], it is possible that the interaction between FSTL1 and other TGF-β superfamily members may also contribute to the role of FSTL1 *in vivo*. Therefore, Fstl1 may have a broad inhibitory effect on BMP signaling pathway by targeting both ligands and receptors.

Our results are consistent with the previous suggestion that BMP signaling regulates ureter development. *Bmp4* heterozygous mutants and *Bmp7* mutants caused defects in urinary tract development. *Bmp7* deficiency caused renal dysplasia and hydroureter phenotype [10], [11],while *Bmp4* heterozygous mutants exhibit multiple defects in urinary system, which is similar with human congenital anomalies of the kidney and urinary tract CAKUT [12]. Indirectly, *Gata2* mutant animals resemble human congenital anomalies of the kidney and urinary tract as a result of reduction in BMP4 abundance [27], [44]. These data indicated reduced BMP signaling is deleterious for urinary tract development. On the other hand, genetic inactivation of the BMP antagonist gremlin 1 (*Grem1*) leads to disruption of metanephric development at the stage of ureteric bud outgrowth initiation [45], indicating BMP antagonists play an essential role in negatively modulating the activity of BMP signals during early kidney development [45], [46]. Our data also add more evidence that precise BMP signaling regulation is important in ureter development after ureteric bud initiation.

Relatively little is known about the mesenchymal-epithelial interaction in ureter development, compared to detailed studies that have focused on the ureteric buds interaction with its surrounding metanephric mesenchymal cells during kidney induction [4], [47]. Recently, it was discovered that Fstl1 was a diffusible mesenchymal factor that determined the epithelium fate during oviduct development [48]. Coincidentally, both *Fstl1* and *Bmp4* are expressed in the ureteral mesenchyme [12]. Our model provides genetic evidence of the existence of signals, such as BMP, from the ureteral mesenchymal layer that affect ureteric epithelium function.

Hydroureter is often associated with cell proliferation and/or apoptosis defects during ureter development in several animal models [8], [12], [13], [31], [33]. In most of these published mouse models, decreased ureteral mesenchymal cell proliferation causes impaired smooth muscle differentiation, finally resulting in a functional obstruction due to defective smooth muscle movement. Although *Fstl1* mRNA is mainly expressed in the ureteral mesenchyme, there were no significant anomalies in proliferation, apoptosis, or differentiation of *Fstl1* null ureteral mesenchyme. On the contrary, the impaired ureteric epithelial proliferation was observed and considered as cause of very narrow distal segment ureters, which makes it difficult for urine to go through, and contributes to hydroureter/ hydronephrosis phenotype.

There is increasing evidence that BMP signaling modulates cell proliferation during development. For instance, overexpressing BMP4 in lung epithelium and hair follicles result in a reduction of proliferation in lung epithelium and the outer root sheath cells in transgenic mouse models [49], [50]. Studies using *in vitro* models suggest that exogenous BMP4 inhibits epithelial cell proliferation and ductal budding in cultured urogenital sinus tissues [51]. Genetic inactivation of *Fstl1* leads to an increased BMP signaling, especially in ureteric epithelium. Consistent with BMP signaling activation, ureteric epithelial cell proliferation was reduced in Fstl1 mutant ureters at E15.5 and E16.5. However, the detail mechanism still needs more studies.

Besides impaired epithelial cell proliferation, *Fstl1^-/-^* ureter also displays defects in subepithelial ureteral mesenchymal cell differentiation. The absence of subepithelial ureteral mesenchymal cells in the *Fstl1^-/-^* ureter is similar with the phenotype in *Shh* mutant ureter. Deletion of *Shh* in the urothelium results in congenital obstructive phenotypes and the subepithelial ureteral mesenchymal cells is missing in this mouse model, suggesting SHH signal plays crucial role in inducing subepithelial ureteral mesenchymal cells differentiation [8]. In *Dlgh1* mutant ureter, the subepithelial ureteral mesenchymal cells which are referred as Raldh2 positive ureteric stromal cells, are also absent. The authors further speculate that this population of cells might provide flexibility during the contraction and relaxation phases of peristalsis, so the missing of these cells might contribute to the hydroureter phenotype [37]. In our study, the time point that subepithelial ureteral mesenchymal cells differentiated and the time point of hydroureter phenotype in *Fstl1^-/-^* to display are also concomitant. Consistent with previous reports suggesting that SHH signaling is crucial for subepithelial ureteral mesenchymal cells differentiation [8], we also observed a down-regulation of SHH signaling in the *Fstl1^-/-^* ureter. Our results indicate that *Fstl1* is required to maintain or establish the subepithelial ureteral mesenchymal cells through at least in part of SHH signal. However, the origin of this cell population is still largely unknown. One possible model is this cell population is derived from the ureteral mesenchymal cells. However, more studies are needed to elucidate the origin and the function of this specific ureteral cell population.

Although we observed that SHH signal is down-regulated in *Fstl1^-/-^* ureter, the hydroureter phenotype of *Fstl1* ureter is more severe than *Shh* conditional mutant in ureteric epithelium. *Fstl1* deficiency caused a down-regulated SHH signal as well as up-regulated BMP signal in urinary tract. Both signals play important regulatory roles in ureter development. The interactions between BMP and SHH signaling are important regulatory mechanisms in multiple developmental processes, including the neural tube patterning, tooth morphogenesis, hair follicle growth induction, limb bud formation, gut development, and left-right determination [52], [53], [54], [55], [56], [57], [58]. Many BMP antagonists regulate the interaction between these two signals [52], [54], [57]. During ureter development, previous reports indicate that SHH signaling could induce Bmp4 activation and promote ureteral mesenchyme proliferation [8]. Our results provide hints that BMP signal could negatively regulate SHH signal, and Fstl1 plays important roles to maintain the balance between these two signaling pathways. However, detailed studies are required to elucidate the exact way that *Fstl1* regulates SHH signal. Anyway, *Fstl1* mutant mice provide a good model to study the mechanisms of the interaction between SHH and BMP signaling pathways to regulate cell proliferation and differentiation during ureter development and in congenital malformations of the urinary tract.

## Supporting Information

Figure S1 ***Fstl1*** **mRNA expression in developing murine ureter.** *Fstl1* whole mount *in situ* hybridization of kidney and ureter at E13.5 (A, B) and E15.5 (C, D). In the cross sections of proximal segments of ureter (B, D), *Fstl1* transcript was detected in ureteral mesenchymal cells (B, D, um), but not in ureteric epithelium at E15.5 (B, D, ue).
(TIF)

Figure S2 ***Fstl1^-/-^*** **embryos developed congenital hydronephrosis.** (A-F) Immunohistochemistry of Pax2 in wild-type (A, C, E) and *Fstl1* ^-/-^ (B, D, F) kidneys at stages of E15.5 (A, B), E16.5 (C, D) and E17.5 (E, F). Note that the size and the collecting duct system of *Fstl1* ^-/-^ kidneys were not affected at E15.5 and E16.5 compared to those of the wild-types (A-D), whereas *Fstl1* ^-/-^ kidney at E17.5 showed hydronephrosis and reduced size (F) compared to wild-type (E). Scale bar: (A-F) 400 µm.
(TIF)

Figure S3 **Defects of UV orifice in** ***Fstl1^-/-^*** **embryo.** (A-D) *Fstl1* ^+/-^ mice were crossed to *Ptch-lacZ* ^+/-^ mice. *Fstl1* ^+/-^; *Ptch-lacZ* ^+/-^ and *Fstl1* ^-/-^; *Ptch-lacZ* ^+/-^ureters were stained for β-galactosidase at E15.5 and E16.5. After sectioned and stained with hematoxylin, histological analysis of the ureterovesical orifice was performed at E15.5 (A, B) and E16.5 (C, D). Note that the distance between the left and right orifices (asterisk) is shorter in the *Fstl1* ^-/-^ embryo (B, D), compared with the wild-type embryo (A, C). (E-H) At E12.5, when the ureter still binds to WD, the length of the CND is similar in both wild-type (E) and *Fstl1^-/-^* (F) embryos. At E12.5, wild-type (G) and *Fstl1^-/-^* (H) CND showed no obvious differences in apoptosis detected by TUNEL assay. (I) Quantification of distance between two ureteral orifices at E15.5 (*p*<0.001, *n* = 7). (J) Quantification of cell apoptosis in CND by TUNEL assay (*p* = 0.29, *n* = 7). Scale bar: (A-D) 100 µm, (E-H) 20 µm. CND: common nephric duct.
(TIF)

Figure S4 **Apoptosis in E15.5 and E16.5 ureter.** Wild-type (A, C) and *Fstl1^-/-^* (B, D) ureters at E15.5 (A, B) and E16.5 (C, D) showed no difference in apoptosis detected by TUNEL assay. Arrows point to representative cells positive for apoptosis. Scale bar: 20 µm.
(TIF)

Figure S5 **Expression of** ***Upk3a*** **was down-regulated in** ***Fstl1^-/-^*** **ureter.** Quantitative real-time PCR of *Upk3a* of E15.5 (n = 6, p = 0.04) and E16.5 (n = 4, *p* = 0.01) ureter.
(TIF)

Figure S6 **Normal ureteral mesenchymal cell differentiation in** ***Fstl1^-/-^*** **ureter.** (A-F) Expression of smooth muscle differentiation markers, α-SMA (A, B), α-SM22 (C, D) and smMHC (E, F) in transverse sections of *Fstl1^-/-^* ureters (B, D, F) shows no obvious difference compared with wild-type ureters (A, C, E) at E15.5. (G) Western blot analysis of smooth muscle differentiation markers. The expression of α-SMA, SM22α and smMHC were not altered in *Fstl1* ^-/-^ ureters at E16.5 compared to Wild-type control. Scale bar: (A-F) 20 µm.
(TIF)

Figure S7 **Subepithelial mesenchymal cells are not detectable at E15.5.** Co-Immunofluorescence staining of α-SMA (green), pan-Cytokeratin (red), and DAPI (blue) in transverse sections of WT ureters (A, C) and *Fstl1^-/-^* ureters at E15.5 (B, D). (C, D) Enlarged views of the boxed area in (A, B). Scale bar: (A, B) 50 µm, (C, D) 10 μm. um: ureteral mesenchyme; ue: ureteric epithelium.
(TIF)

Figure S8 **Upregulation of phosphorylated Smad1/5/8 level in** ***Fstl1^-/-^*** **ureter.** pSmad1/5/8 immunohistochemistry on transverse sections from WT (A, C, E) and *Fstl1* ^-/-^ (B, D, F) ureters at E15.5 (A, B), E16.5 (C, D) and E18.5 (E, F). Note that pSmad1/5/8 staining was stronger in the *Fstl1^-/-^* ureter (B, D, F). Scale bar: 40 µm.
(TIF)

Figure S9 **Normal TGF-β signal in** ***Fstl1*** **^-/-^ kidney and ureter.** Western blots of pSmad2 for E18.5 kidney protein (left panels), and E15.5 ureter protein (right panels).
(TIF)

Figure S10 **Fstl1 can antagonize BMP4/BMP2-induced stimulation** ***in vitro*** **.** (A) Western blots of pSmad1/5/8, Smad1, pAKT (Ser^473^), AKT, GAPDH of HEK293 cells treated by adding BMP4 (20 ng/ml) and BMP2 (10 ng/ml) the conditional media transfected either by Fstl1 or pcDNA3.1 vector (Mock) for 30min. (B) Co-immunoprecipitation of Myc-Fstl1 and HA-tagged BMP type I receptors in COS7 cells. Myc-Fstl1 can be immunoprecipitated with the anti-c-Myc antibody. Note that both HA-ALK6 and HA-ALK3 were co-immunoprecipitated by the anti-c-Myc and detected by the anti-HA antibody (lane 4, 6).
(TIF)

Table S1 **primers for** ***in situ*** **probe and real time PCR.**
(DOC)

Movie S1 **Ureteral peristalsis in a wild-type ureter.**
(MP4)

Movie S2 **Ureteral peristalsis in an** ***Fstl1*** **^-/-^ ureter.**
(MP4)
