# Supplementary figures and images for: Fstl1 Antagonizes BMP Signaling and Regulates Ureter Development
Source: PLoS One. 2012 Apr 2;7(4):e32554. doi: 10.1371/journal.pone.0032554 (PMC3317656; doi:10.1371/journal.pone.0032554)

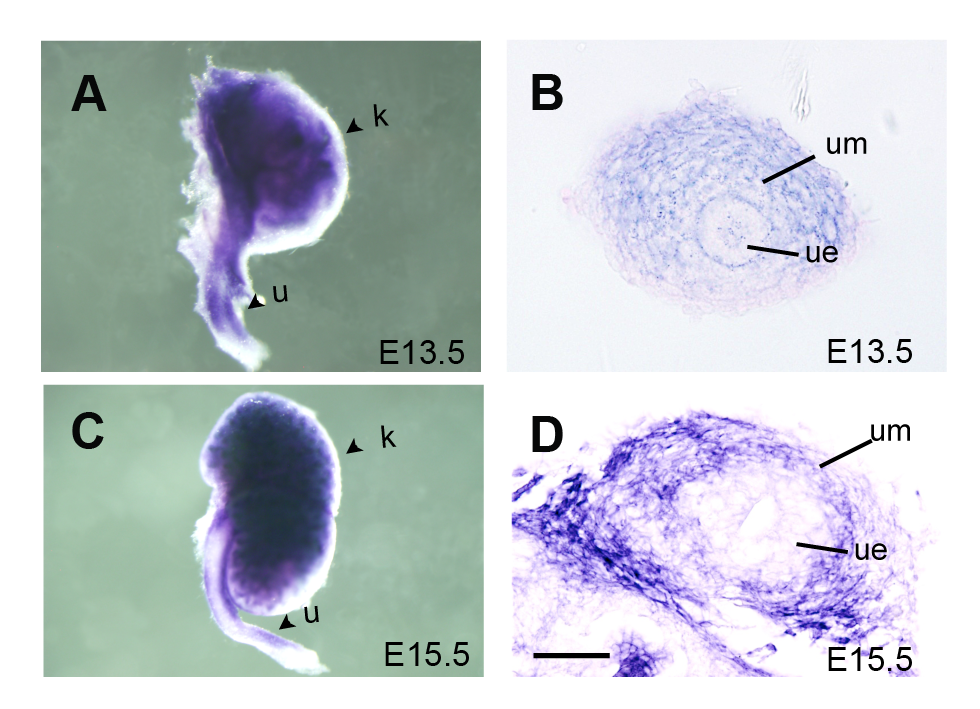

Supplement: Figure S1 — Fstl1 mRNA expression in developing murine ureter. Fstl1 whole mount in situ hybridization of kidney and ureter at E13.5 (A, B) and E15.5 (C, D). In the cross sections of proximal segments of ureter (B, D), Fstl1 transcript was detected in ureteral mesenchymal cells (B, D, um), but not in ureteric epithelium at E15.5 (B, D, ue). (TIF) [file pone.0032554.s001.tif]

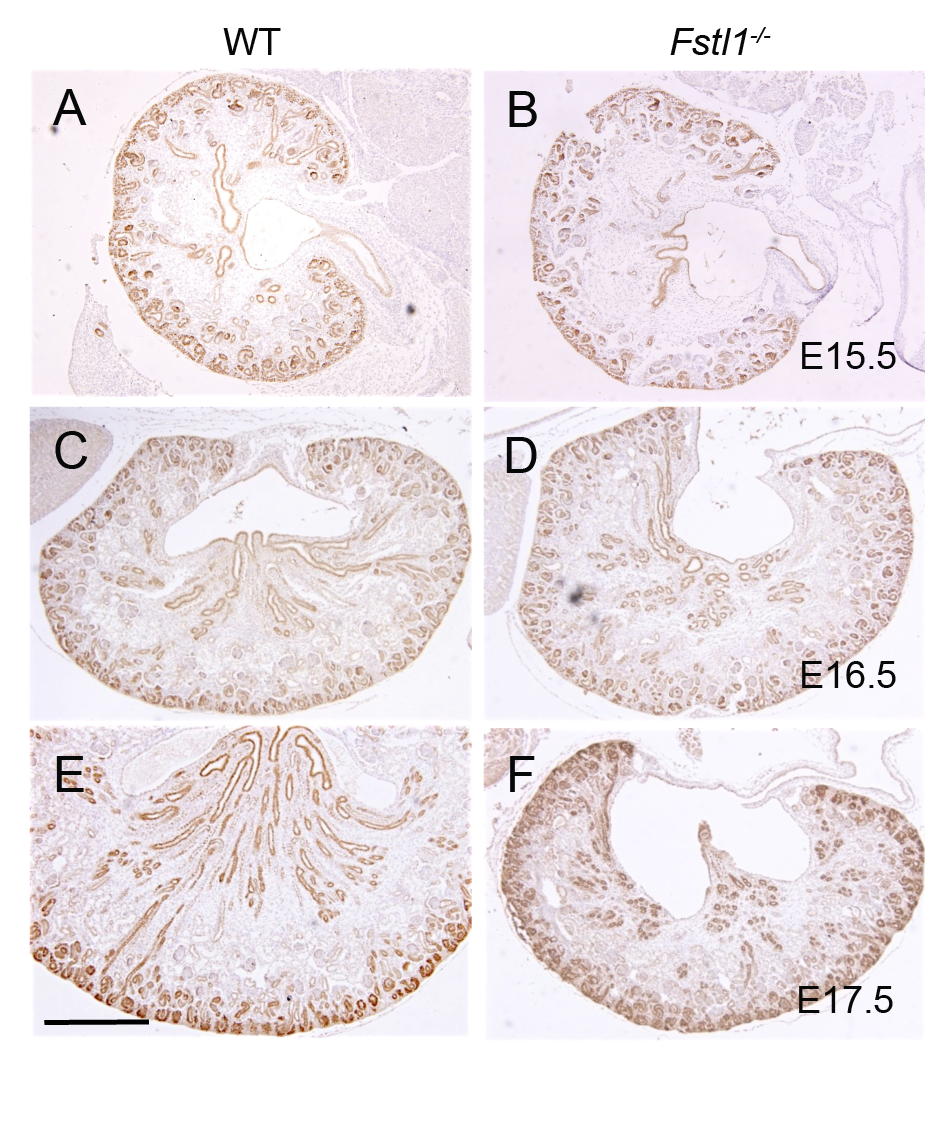

Supplement: Figure S2 — Fstl1-/- embryos developed congenital hydronephrosis. (A-F) Immunohistochemistry of Pax2 in wild-type (A, C, E) and Fstl1 -/- (B, D, F) kidneys at stages of E15.5 (A, B), E16.5 (C, D) and E17.5 (E, F). Note that the size and the collecting duct system of Fstl1 -/- kidneys were not affected at E15.5 and E16.5 compared to those of the wild-types (A-D), whereas Fstl1 -/- kidney at E17.5 showed hydronephrosis and reduced size (F) compared to wild-type (E). Scale bar: (A-F) 400 µm. (TIF) [file pone.0032554.s002.tif]

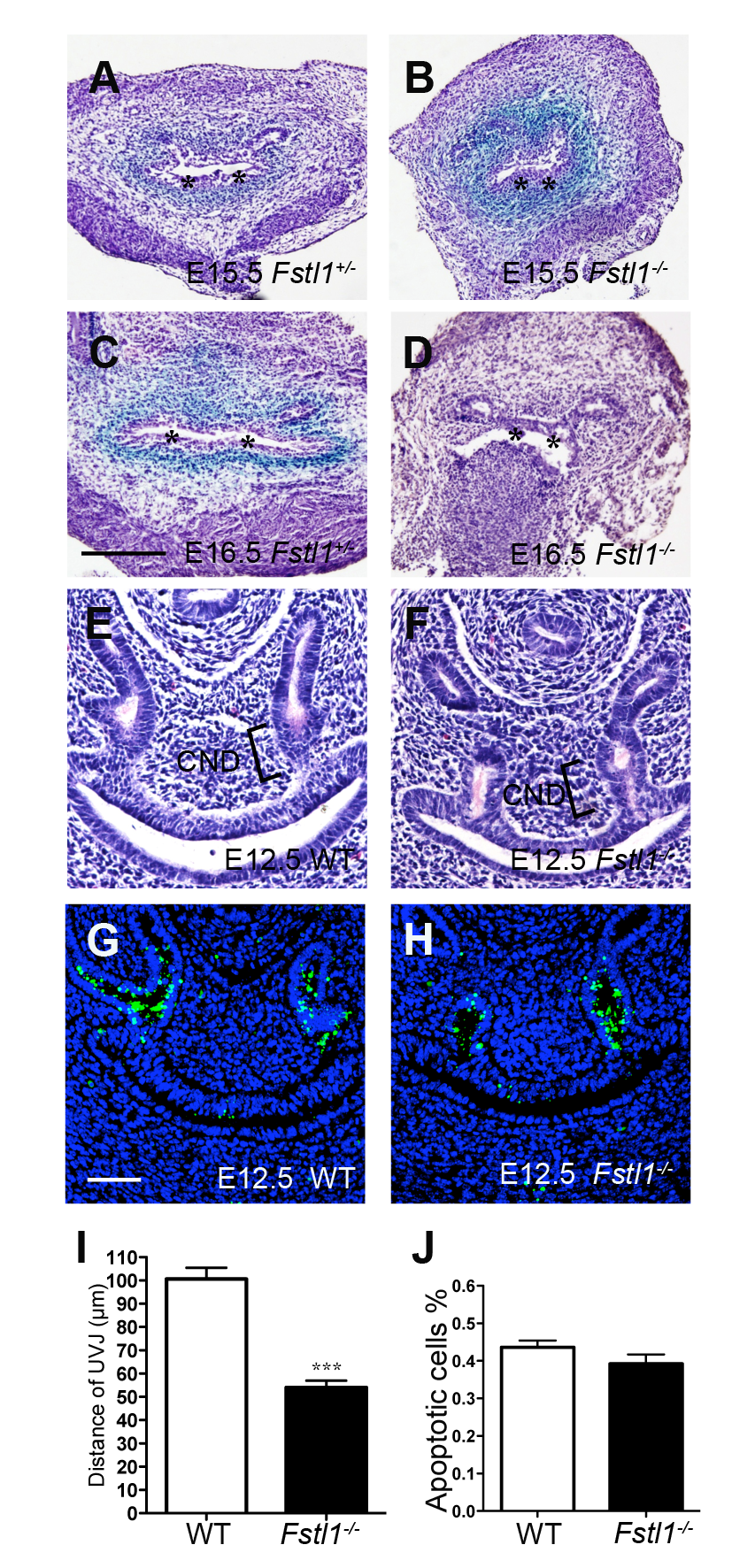

Supplement: Figure S3 — Defects of UV orifice in Fstl1-/- embryo. (A-D) Fstl1 +/- mice were crossed to Ptch-lacZ +/- mice. Fstl1 +/-; Ptch-lacZ +/- and Fstl1 -/-; Ptch-lacZ +/-ureters were stained for β-galactosidase at E15.5 and E16.5. After sectioned and stained with hematoxylin, histological analysis of the ureterovesical orifice was performed at E15.5 (A, B) and E16.5 (C, D). Note that the distance between the left and right orifices (asterisk) is shorter in the Fstl1 -/- embryo (B, D), compared with the wild-type embryo (A, C). (E-H) At E12.5, when the ureter still binds to WD, the length of the CND is similar in both wild-type (E) and Fstl1-/- (F) embryos. At E12.5, wild-type (G) and Fstl1-/- (H) CND showed no obvious differences in apoptosis detected by TUNEL assay. (I) Quantification of distance between two ureteral orifices at E15.5 (p<0.001, n = 7). (J) Quantification of cell apoptosis in CND by TUNEL assay (p = 0.29, n = 7). Scale bar: (A-D) 100 µm, (E-H) 20 µm. CND: common nephric duct. (TIF) [file pone.0032554.s003.tif]

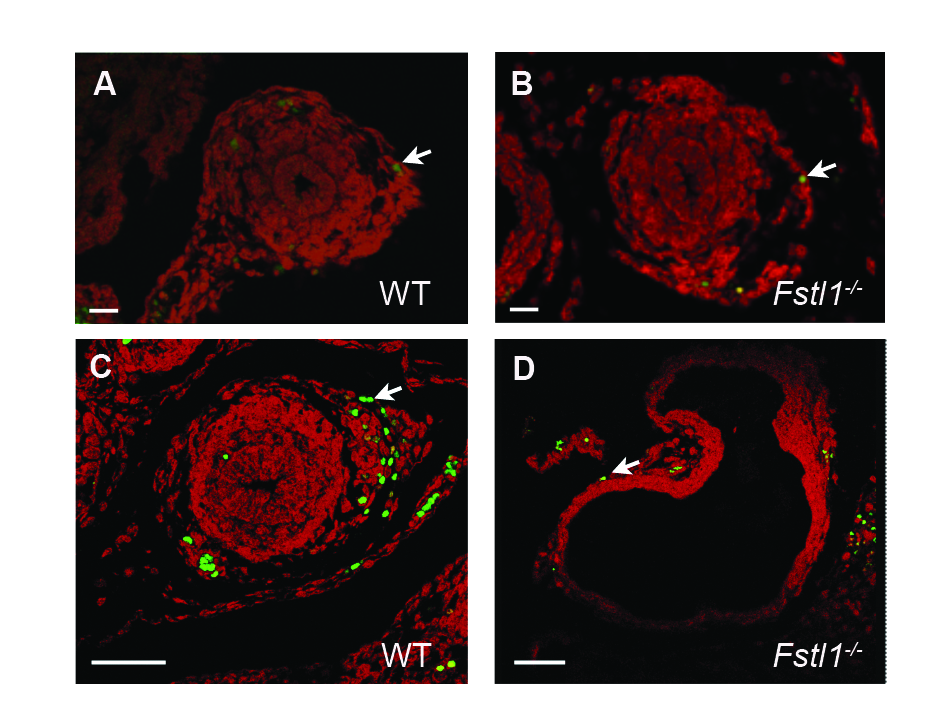

Supplement: Figure S4 — Apoptosis in E15.5 and E16.5 ureter. Wild-type (A, C) and Fstl1-/- (B, D) ureters at E15.5 (A, B) and E16.5 (C, D) showed no difference in apoptosis detected by TUNEL assay. Arrows point to representative cells positive for apoptosis. Scale bar: 20 µm. (TIF) [file pone.0032554.s004.tif]

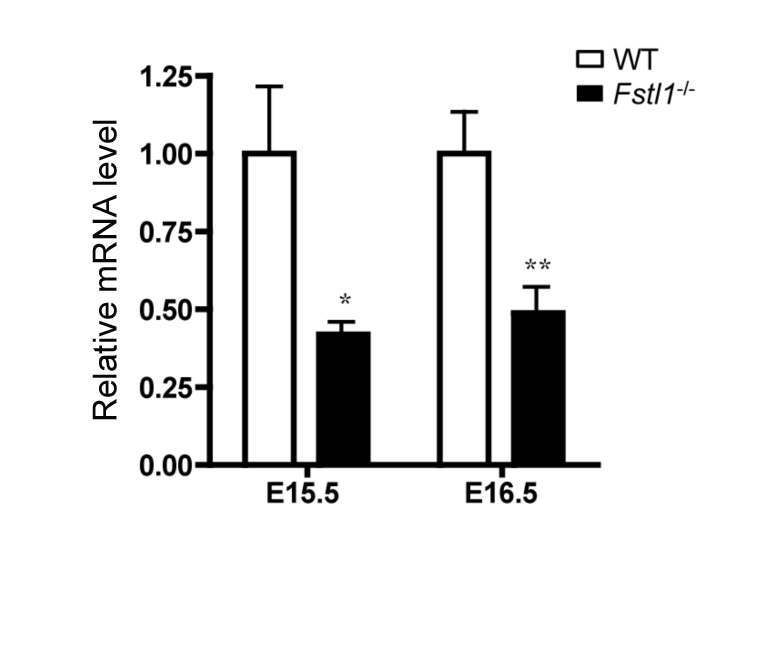

Supplement: Figure S5 — Expression of Upk3a was down-regulated in Fstl1-/- ureter. Quantitative real-time PCR of Upk3a of E15.5 (n = 6, p = 0.04) and E16.5 (n = 4, p = 0.01) ureter. (TIF) [file pone.0032554.s005.tif]

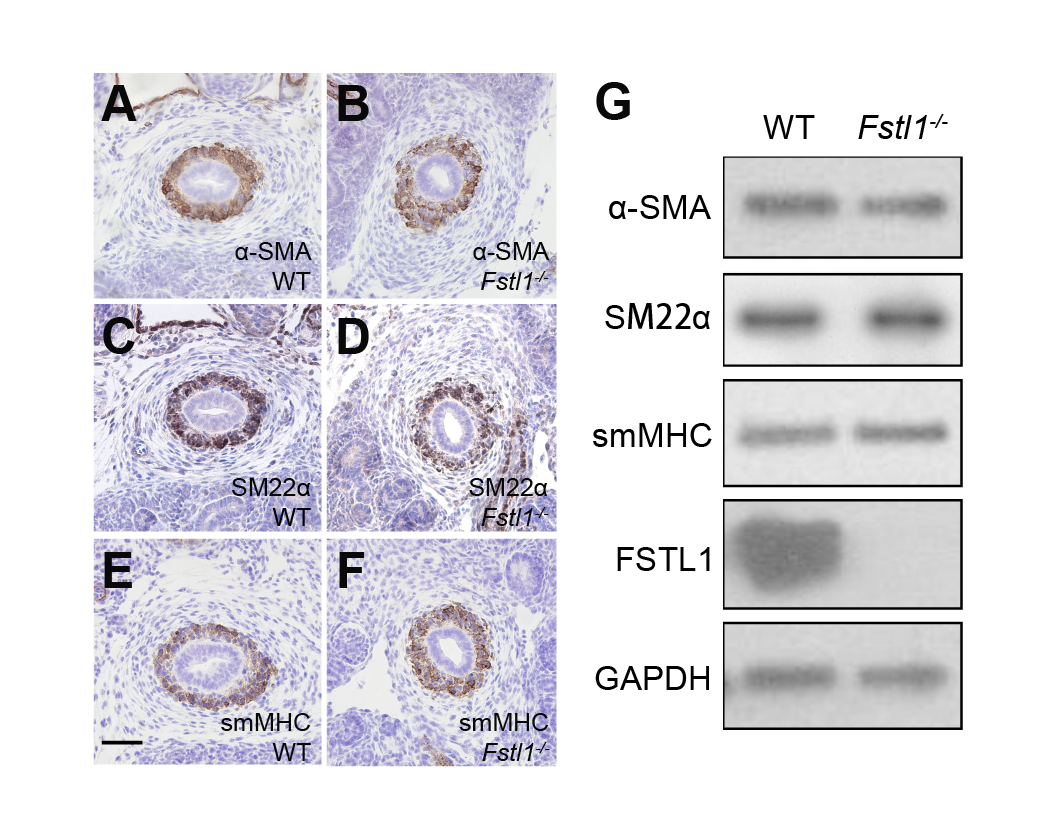

Supplement: Figure S6 — Normal ureteral mesenchymal cell differentiation in Fstl1-/- ureter. (A-F) Expression of smooth muscle differentiation markers, α-SMA (A, B), α-SM22 (C, D) and smMHC (E, F) in transverse sections of Fstl1-/- ureters (B, D, F) shows no obvious difference compared with wild-type ureters (A, C, E) at E15.5. (G) Western blot analysis of smooth muscle differentiation markers. The expression of α-SMA, SM22α and smMHC were not altered in Fstl1 -/- ureters at E16.5 compared to Wild-type control. Scale bar: (A-F) 20 µm. (TIF) [file pone.0032554.s006.tif]

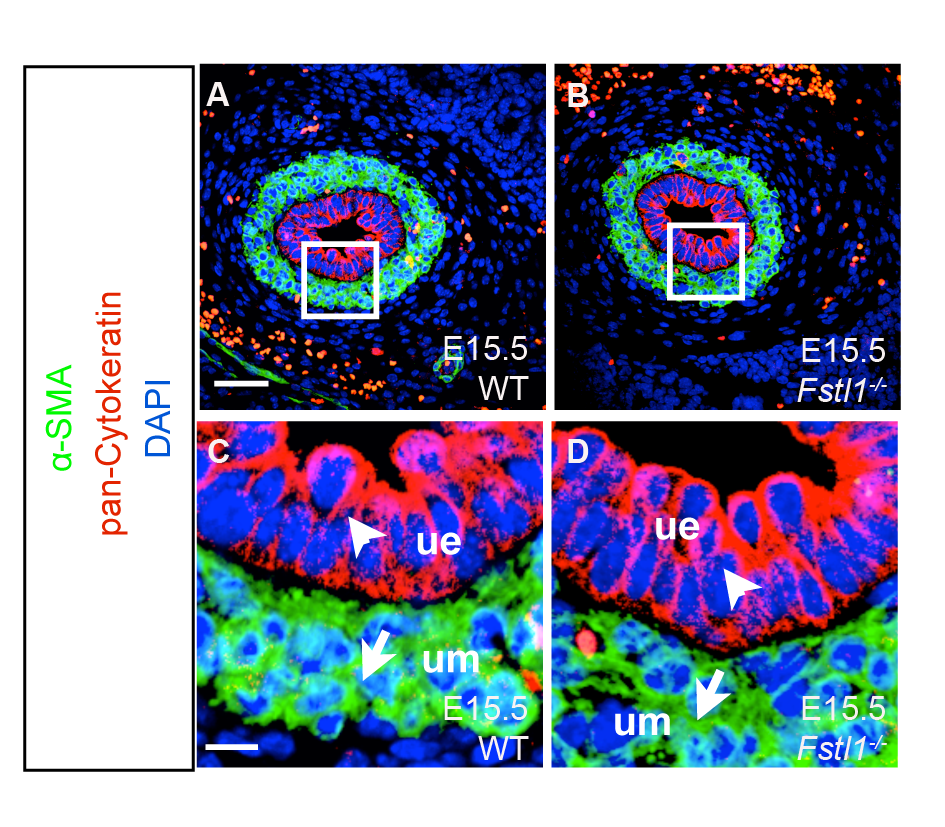

Supplement: Figure S7 — Subepithelial mesenchymal cells are not detectable at E15.5. Co-Immunofluorescence staining of α-SMA (green), pan-Cytokeratin (red), and DAPI (blue) in transverse sections of WT ureters (A, C) and Fstl1-/- ureters at E15.5 (B, D). (C, D) Enlarged views of the boxed area in (A, B). Scale bar: (A, B) 50 µm, (C, D) 10 μm. um: ureteral mesenchyme; ue: ureteric epithelium. (TIF) [file pone.0032554.s007.tif]

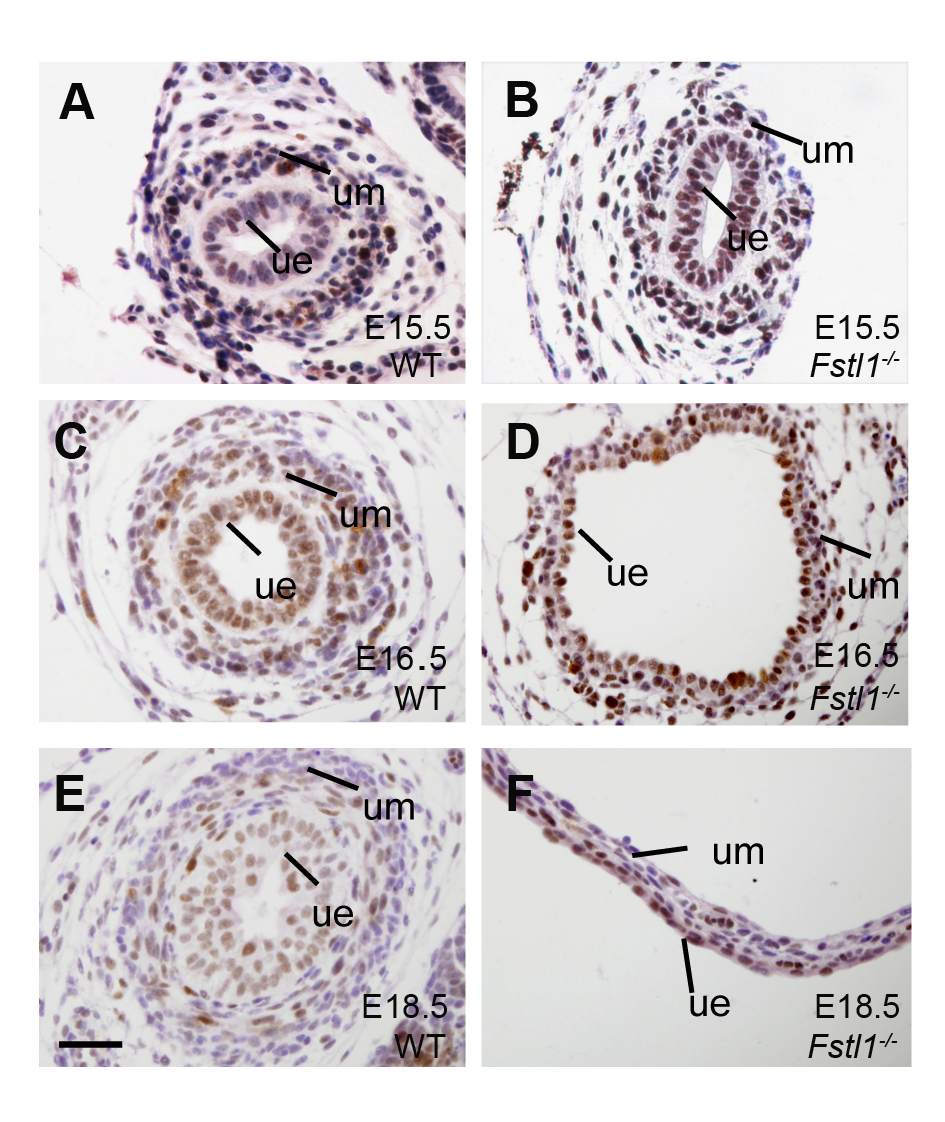

Supplement: Figure S8 — Upregulation of phosphorylated Smad1/5/8 level in Fstl1-/- ureter. pSmad1/5/8 immunohistochemistry on transverse sections from WT (A, C, E) and Fstl1 -/- (B, D, F) ureters at E15.5 (A, B), E16.5 (C, D) and E18.5 (E, F). Note that pSmad1/5/8 staining was stronger in the Fstl1-/- ureter (B, D, F). Scale bar: 40 µm. (TIF) [file pone.0032554.s008.tif]

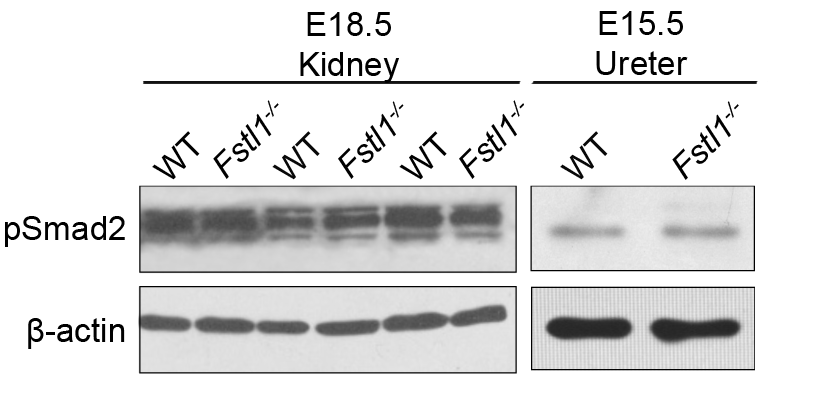

Supplement: Figure S9 — Normal TGF-β signal in Fstl1 -/- kidney and ureter. Western blots of pSmad2 for E18.5 kidney protein (left panels), and E15.5 ureter protein (right panels). (TIF) [file pone.0032554.s009.tif]

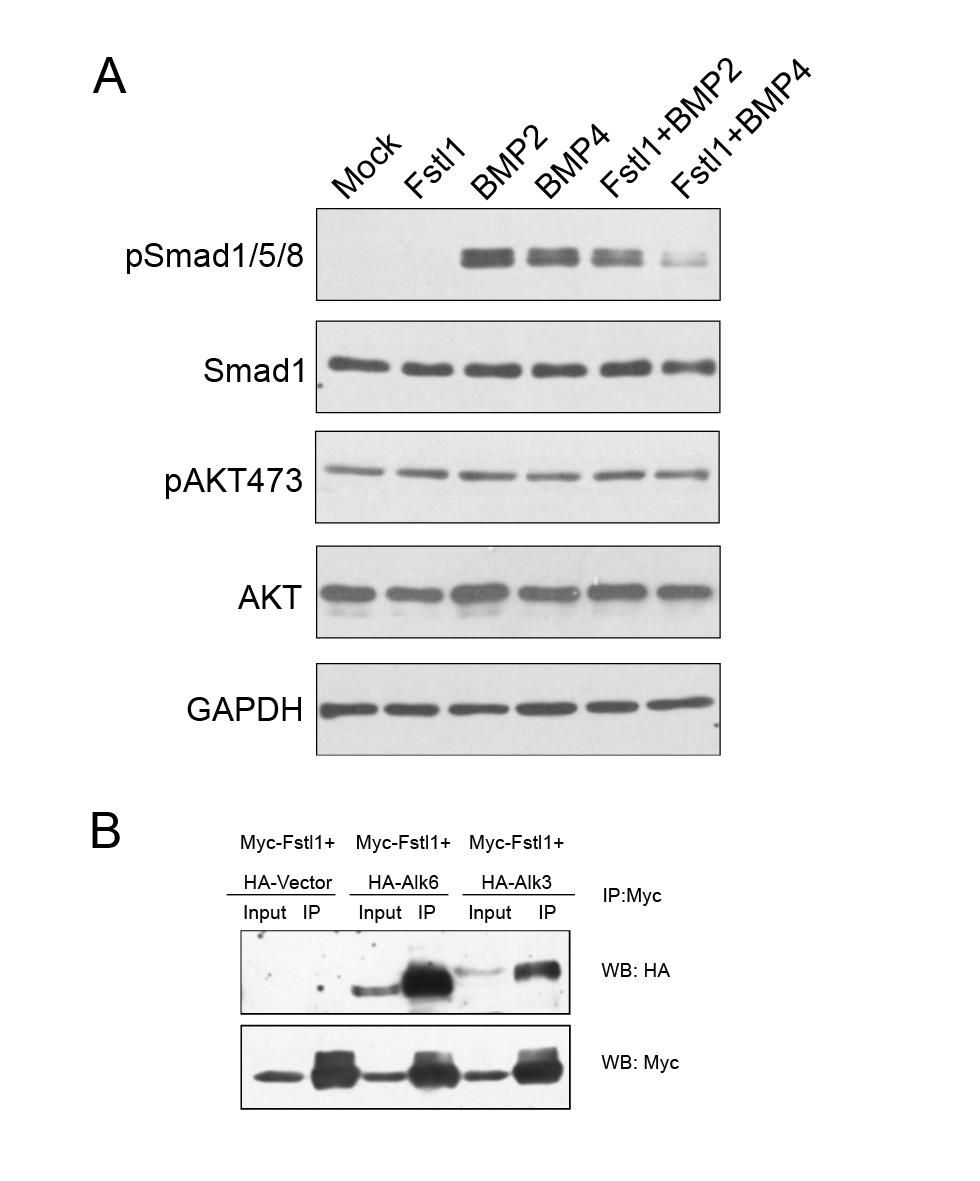

Supplement: Figure S10 — Fstl1 can antagonize BMP4/BMP2-induced stimulation in vitro . (A) Western blots of pSmad1/5/8, Smad1, pAKT (Ser473), AKT, GAPDH of HEK293 cells treated by adding BMP4 (20 ng/ml) and BMP2 (10 ng/ml) the conditional media transfected either by Fstl1 or pcDNA3.1 vector (Mock) for 30min. (B) Co-immunoprecipitation of Myc-Fstl1 and HA-tagged BMP type I receptors in COS7 cells. Myc-Fstl1 can be immunoprecipitated with the anti-c-Myc antibody. Note that both HA-ALK6 and HA-ALK3 were co-immunoprecipitated by the anti-c-Myc and detected by the anti-HA antibody (lane 4, 6). (TIF) [file pone.0032554.s010.tif]
