# Supplementary material for: Fstl1 Antagonizes BMP Signaling and Regulates Ureter Development
Source: PLoS One. 2012 Apr 2;7(4):e32554. doi: 10.1371/journal.pone.0032554 (PMC3317656; doi:10.1371/journal.pone.0032554)
Supplement: Table S1 — primers for in situ probe and real time PCR. (DOC) [file pone.0032554.s011.doc]

**Table S1.** primers for *in situ* probe and real time PCR

| FRP probe-5p | ATCCTTTCTTTGAGCCTCCG |
| --- | --- |
| FRP probe-3p | TGTAATCCTGCGTGTTGGTC |
| Bmp2-sybr-f | CCTCAAGTCCAGCTGCAAGAG |
| Bmp2-sybr-r | GGTGCCACGATCCAGTCATT |
| Bmp4-sybr-f | GCACTGCCGCAGCTTCTC |
| Bmp4-sybr-r | CACTGACAGAAAACAAGGCATATAATAA |
| Bmp7-sybr-f | ACCCTTCATGGTGGCCTTCT |
| Bmp7-sybr-r | CCTCAGGGCCTCTTGGTTCT |
| Tgfb1-sybr-f | TGGCGTTACCTTGGTAACC |
| Tgfb1-sybr-r | GGTGCTGGGCCCTTTCCAG |
| Shh-sybr-f | AGACCGGCTGATGACTCAGA |
| Shh-sybr-r | CGACCCTCATAGTGTAGAGAC |
| b-actin-sybr-f | GAGAAGATCTGGCACCACACC |
| b-actin-sybr-r | GCATACAGGGACAGCACAGC |
| bmpr1aqPCRf | GAAGTTGCTGTATTGCTGA |
| bmpr1aqPCRr | GTAATACAACGACGAGCC |
| bmpr1bqPCRf | ATACCAGCTTCCCTATCACGACCT |
| bmpr1bqPCRr | TGAAATTCTTGCTCTGTCCACAAGTA |
| bmpr2qPCRf | AGGATCAGGTGAAAAGATCAAGAGA |
| bmpr2qPCRr | GCAAGGTACACAGCAGTGCTAGATT |
| tgfbr1qPCRf | ATCTATGCAATGGGCTTAGTGTTCT |
| tgfbr1qPCRr | GCCTTAACTTCTGTTCGCAAACTACT |
| ActRIIaqPCRf | TCTTGATATAGGTTTGCTTGCACTTG |
| ActRIIaqPCRr | CCGAACAGTCGTATTATCCCTCATT |
| ActRIIbqPCRf | CAGGGACTTCAAAAGCAAGAATGT |
| ActRIIbqPCRr | AGGAAGGCGTCTCTCTGGAAGTT |
